# Tunable Single-Photon Emission with Wafer-Scale Plasmonic Array

**DOI:** 10.1021/acs.nanolett.3c05155

**Published:** 2024-02-15

**Authors:** Chun-An Chen, Po-Han Chen, Yu-Xiang Zheng, Chiao-Han Chen, Mong-Kai Hsu, Kai-Chieh Hsu, Ying-Yu Lai, Chih-Sung Chuu, Hui Deng, Yi-Hsien Lee

**Affiliations:** † Department of Materials Science and Engineering, National Tsing Hua University, Hsinchu 30013, Taiwan; ‡ Department of Physics, University of Michigan, Ann Arbor, Michigan 48109-2122, United States; § Department of Physics, National Tsing Hua University, Hsinchu 30013, Taiwan

**Keywords:** plasmonic, single crystal, single-photon emitter, hexagonal boron nitride

## Abstract

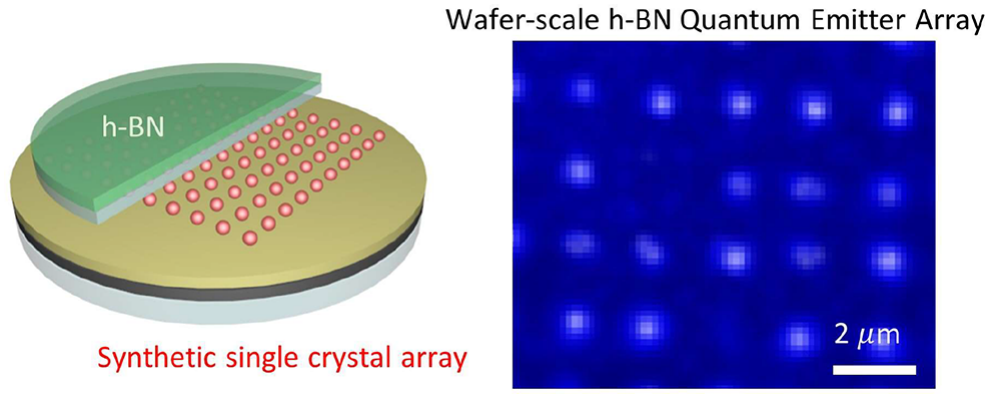

Bright, scalable, and deterministic single-photon emission (SPE) is essential for quantum optics, nanophotonics, and optical information systems. Recently, SPE from hexagonal boron nitride (h-BN) has attracted intense interest because it is optically active and stable at room temperature. Here, we demonstrate a tunable quantum emitter array in h-BN at room temperature by integrating a wafer-scale plasmonic array. The transient voltage electrophoretic deposition (EPD) reaction is developed to effectively enhance the filling of single-crystal nanometals in the designed patterns without aggregation, which ensures the fabricated array for tunable performances of these single-photon emitters. An enhancement of ∼500% of the SPE intensity of the h-BN emitter array is observed with a radiative quantum efficiency of up to 20% and a saturated count rate of more than 4.5 × 10^6^ counts/s. These results suggest the integrated h-BN-plasmonic array as a promising platform for scalable and controllable SPE photonics at room temperature.

Single-photon emission (SPE) from materials, including diamond color center,^1^ SiC,^2,3^ III–V semiconductor quantum dots (QD),^4^ and van der Waals (vdW) materials,^5,6^ is significant for next-generation communications and computing. Many of these materials require cryogenic temperatures for SPE to trap the emitters or suppress phonon sidebands. SPE operated at elevated temperatures has been realized in epitaxial III-Nitride semiconductors,^7−12^ but the emitters need to be embedded inside a III-Nitride host material, which limits further integration with photonic systems. Recently, stable SPE from hexagonal boron nitride (h-BN) has been demonstrated at room temperature (RT) with high quantum efficiencies and a high Debye–Waller factor with emitters naturally embedded in an atomically thin 2D lattice.^13−16^ However, there remain long-lasting challenges in scaling and deterministic SPE of the h-BN emitters. The SPE generally shows brightness determined by the SPE quantum efficiency and it is limited by the SPE lifetime of several nanoseconds. Considerable efforts have been devoted to realize controllable SPE brightness and lifetime via nanofabrication, development of synthetic emitters, and defects engineering.^17−23^ One of the most promising routes is based on plasmonic effects by heterogeneous integration with metallic nanostructures.^24^ An array of nanoantennae with wafer-scale uniformity enables coupling between plasmonic surface resonance states and defect levels in h-BN,^18,19^ which leads to a decrease of the SPE radiative lifetime and, thereby, an increase of the zero-phonon line (ZPL) intensity of the SPE.^17−19,25−28^ The SPE enhancement is highly sensitive to the quality and control of the plasmonic array because it depends on the coupling between the SPE and plasmonic array, as well as the contamination introduced in the heterogeneous integration process.^29−31^ Most reported studies adopted top-down fabrication on the basis of evaporated metals because the process is relatively simple and robust in size control of designed patterns. For conventional plasmonic nanostructures with high field enhancement,^32,33^ larger optical losses commonly appear because of scattering among electrons by defects and grain boundaries formed in individual and nanosized metals.

To reduce the loss and improve the control of the size of the plasmonic structures, the bottom-up route based on single-crystalline metallic nanostructures was proposed, and well-controlled spatial distribution of single-crystalline units was achieved but with mesostructured metals of size over 100 nm.^34,35^ While integrating the synthetic 2D emitters with the plasmonic array allows effective control of emission properties on a wafer scale, it is not experimentally achieved because of two reasons. First, it requires precise control of the shape and size of sub-100 nm units while maintaining their quality as single crystals for the resonance to effectively enhance the ZPL state of h-BN emitters. Second, it is challenging to achieve spatial control over the artificial assembly patterns and ensure reliable filling of sub-100 nm metallic units.^34,35^ At an isotropic geometry of the metallic unit with dimensions below 100 nm, the plasmon resonance peak typically manifests between 520 and 600 nm, which is ideal for the h-BN SPE.

Here, we demonstrate the artificial plasmonic arrays of assembled sub-100 nm single-crystalline metal units and scalable fabrication of RT quantum emitters with synthesized h-BN. The long-lasting challenges of SPE photonics, including scaling, brightness, and deterministic and tunable SPE at room temperature, are studied and overcome by integrating our synthetic h-BN and the fabricated plasmonic array. The scalable SPE exhibits a radiative quantum efficiency of up to 20% and a saturated count rate in excess of 4.5 × 10^6^ counts/s. Possible routes to fabricate artificial patterns of the assembled single-crystal units are illustrated for controllable properties in SPE photonics.

## Wafer-Scale Quantum Emitters Array of h-BN at RT

**Figure 1:**
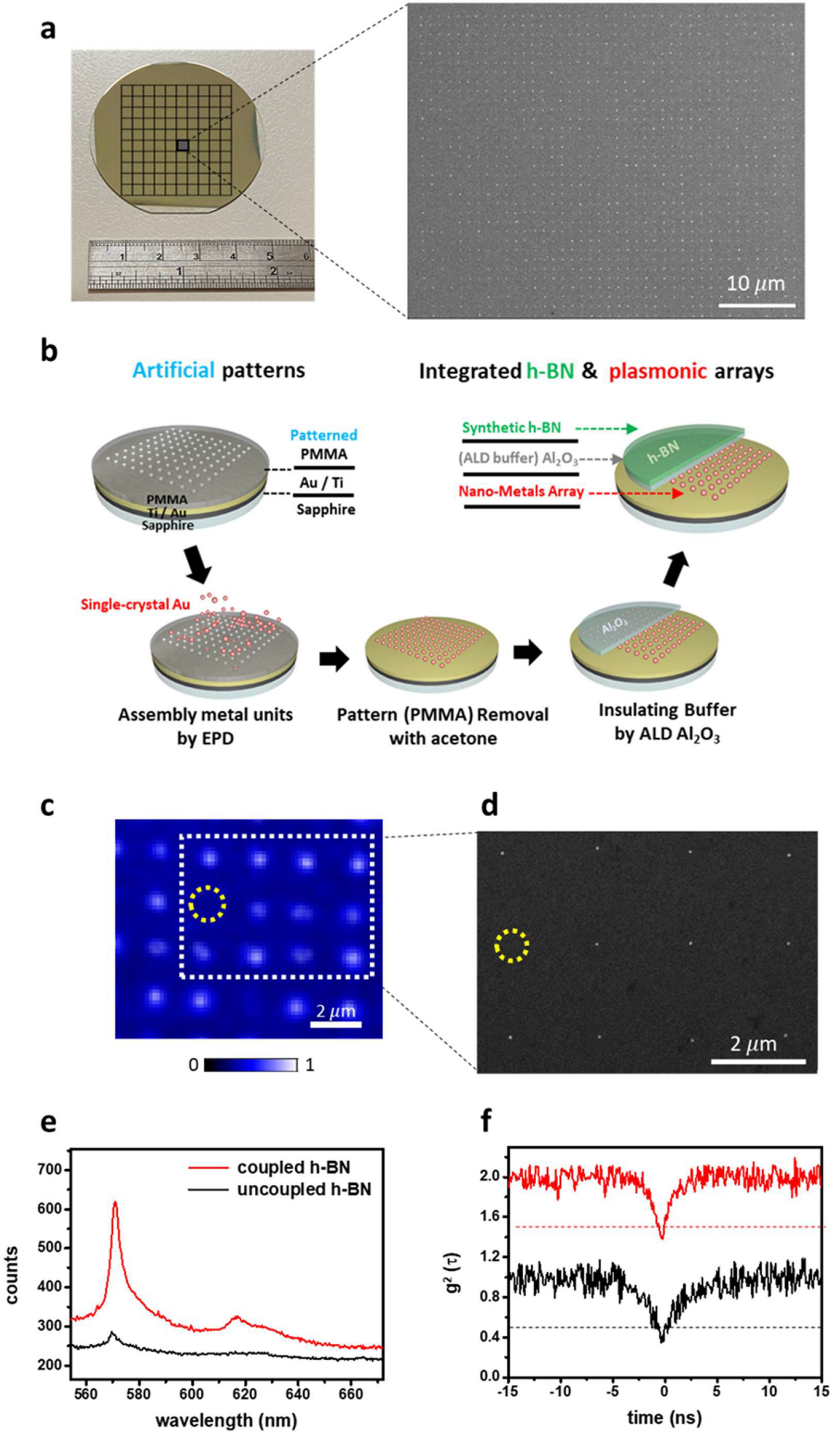
Wafer-scale quantum emitters array of h-BN at RT. (a) Wafer map of the integrated h-BN emitters and array with an SEM image for an enlarged view of the fabricated array. (b) Schematic of the wafer fabrication of the artificial plasmonic array with the h-BN quantum emitters. (c) PL mapping image for the ZPL emissions of the integrated h-BN and the NS array. The PL map was obtained by integrating the luminescence for the spectral range from 560 to 580 nm. (d) The magnified SEM image on the array of the assembled monomers. (e) PL spectrum and (F) the second-order correlation function of the uncoupled (black curve) and the coupled (red curve) SPE.

A wafer-scale quantum emitters array is realized by integrating the synthesized h-BN with a designed array of single-crystalline nanostructures, as shown in Figure 1. Optical and scanning electron microscope (SEM) images of the wafer (Figure 1a and Figure S1) show regular nanosphere (NS) arrays of precisely controlled positions. We choose the geometrically isotropic NS of the gold single crystal as a fundamental unit for the nanostructure array because it allows robust control of the size, surface configurations, and properties of the synthesized metals. The seed-mediated growth enables monodispersity of the synthesized NS single crystals of diameters ranging from 9 to 104 nm, as shown in Figure S2.^36−38^Figure 1b presents a schematic illustration of the fabrication of the designed NS arrays. The wafer-level fabrication involves electron beam lithography of the patterned holes with a resolution of 10–20 nm, mass production of the geometrically tuned nanometals, assembly of the synthesized single-crystal NS by the electrophoretic deposition (EPD) process, and integration of the h-BN on a 2 in. sapphire wafer. To avoid quenching of the SPE, a thin insulating layer of the Al_2_O_3_ (5 nm) was deposited on the array before integration with the h-BN. The large-area h-BN was synthesized by low-pressure chemical vapor deposition (LPCVD) with the random distribution of high-density emitters featuring a narrow ZPL bandwidth of about 100 meV (Figures S3–S5), which is consistent with reported studies.^39−41^ In Figure 1c, the confocal spectral mapping of PL shows enhanced emission at the sites of every Au-NS, which is manifested as a brighter spot in the PL map. In contrast, a uniform but weak emission appears in the area without the assembled NS (circled with a yellow marker in Figure 1c,d). The enhanced emission by the NS is further illustrated in the emission spectrum, as shown in Figure 1e. The ZPL emission of the h-BN is at 570 nm, and a phonon sideband is at around 620 nm, which is consistent with a recent paper.^42^ The different brightnesses among the coupled emitters at different NS sites are due to variations in the spatial distributions of the emitters at the different NSs. In Figure 1d, the SEM image displays the morphology of the monomer array to highlight the missing NS for the reduced SPE. In the Figure S6a, the AFM image and line-scan profile of the height confirm that the synthesized NS assembled in the array exhibits single-crystalline signatures with uniform shape and size (diameter = ∼60 nm) in the whole wafer. The array of the assembled polycrystalline NS is deposited with conventional e-gun evaporation for comparison (Figure S6b). Figure 1f presents the second-order correlation measurements on the coupled (red) and the uncoupled (black) emitters. The *g*^2^(0) of the uncoupled and naturally isolated emitters is ∼0.4, while it is reduced to 0.34 for the emitter coupled with an NS. This confirms single-photon emission from the h-BN emitters and improved single-photon purity by NS (Figure S7).

## Scalable Fabrication of Single-Crystalline Nanostructure Array

**Figure 2:**
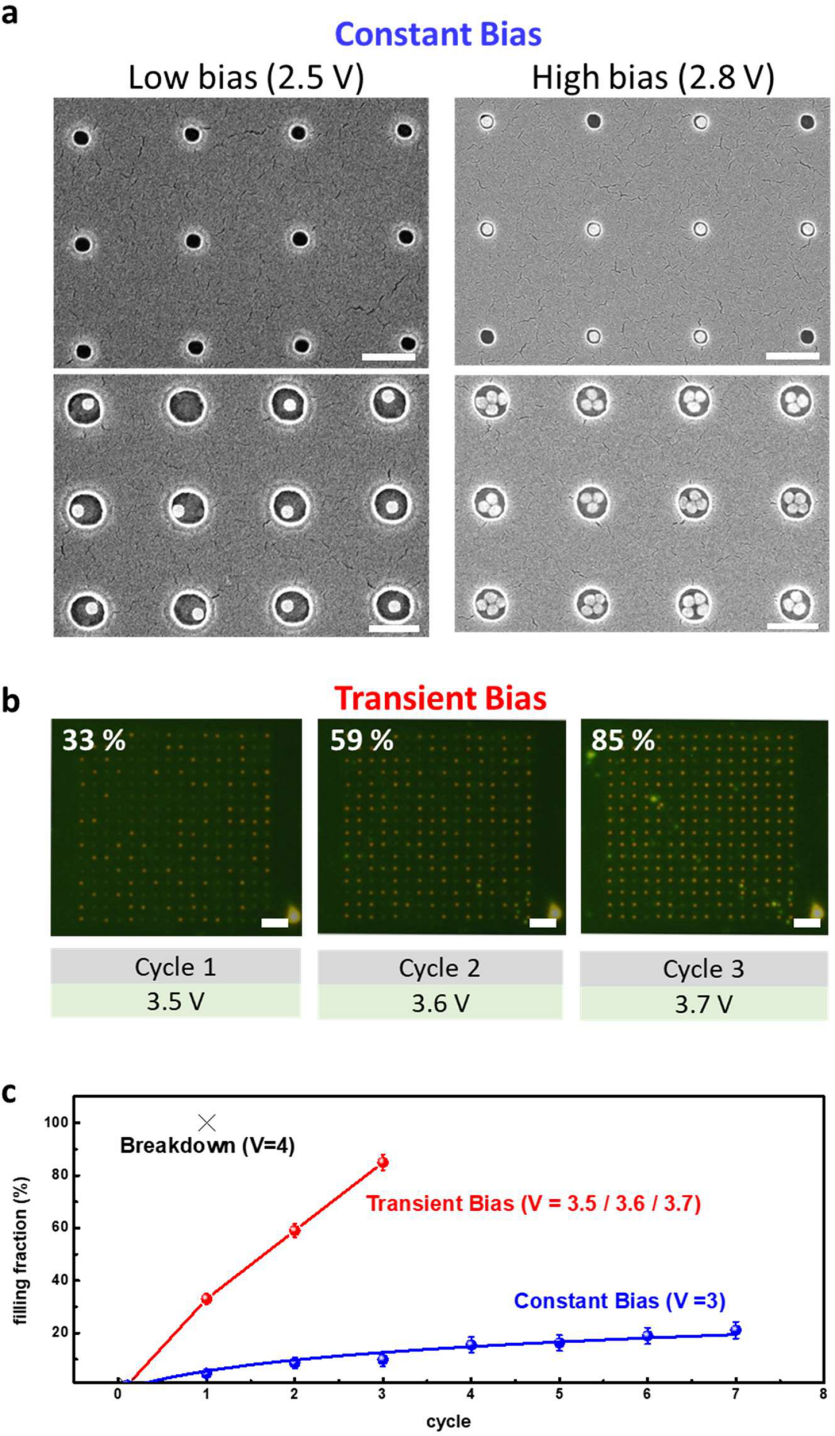
Scalable fabrication of single-crystalline nanostructure array. (a) Constant-bias SEM images of EPD reactions with large NS (scale bar: 500 nm). (b) Transient-bias dark-field images of repeated EPD reactions featuring small NS with the increasing voltage from 3.5 to 3.7 V (scale bar: 2 μm). (c) Filling fraction of gold NS under the voltage of constant 3 V, constant 4 V, and increasing voltage.

To realize the scaling and deterministic properties of the SPE array, a scalable fabrication for a designed array of assembled single crystals with high filling fraction and uniformity is required. The EPD of the functionalized nanomaterials is suitable for the fabrication of designed patterns.^34,35^ However, previous EPD-based arrays have had NS units of a size larger than 100 nm because of critical issues on filling fraction, damages of the pattern, and nonuniform surface charge of the unit for suspension in EPD reactions. Filling of sub-100 nm NSs remains challenging because of two reasons. First, the lower surface charge of smaller nanoparticles induces weaker Coulomb forces than the force in Brownian motion. Second, filling the Au NS in a reduced hole size requires a larger electric field. To illustrate this issue, we have created nanopatterns with pore sizes of 120 and 300 nm filled with Au NSs of 110 nm and operated at a constant voltage of 2.5 and 2.8 V. At 2.5 V, the fraction of deposited 300 nm holes was found to be 90% (Figure 2a). Conversely, for the 120 nm hole, no deposition was observed at 2.5 V, but an increased filling fraction of 67% was identified at a higher voltage of 2.8 V.

To create an enhanced SPE array in h-BN, here, we create NS arrays with reduced sizes of the hole patterns and synthesized NSs of small diameters of 40–60 nm. Figure S8 shows bias-dependent assembling of the 60 nm NSs in the EPD reactions, thereby indicating that filling of the NS into the hole patterns is clearly improved (from 4% to 60%) with the applied voltages. However, possible damages of the hole pattern or aggregations of the NS appear at a voltage higher than 3.7 V. To increase the filling fraction of the assembling, cycling of the EPD reactions was carried out. Figure S9 presents cycling-dependent assembly in EPD reactions at constant voltages with an operation time of 60 s for each cycle. Note that in the assembling at the low bias of 3 V, the filling is improved (from 4% to 21%) but remains a low fraction after seven cycles of the EPD reactions. At a high applied voltage of 3.9 V, aggregation of the Au-NS occurs in the EPD reactions, and most of the holes were filled with the aggregated NS (Figure S8). Moreover, considerable damage to the polymer layer for the designed patterns (on the sapphire substrate) appears with the applied voltages higher than 4 V, and more details were shown in Figure S9. To overcome the above issues, a modified route based on the variable bias (the increasing voltage) is found to effectively enhance the filling in the EPD reactions without aggregation of the NS and damage of the hole pattern of the polymer layer, as shown in (Figure 2b). Under weak electric fields (3.5 V), nanocrystals have lower kinetic energy and can be deposited with high selectivity into our designed conductive nanoholes. With the variable bias EPD reactions (3.5, 3.6, and 3.7 V), the filling of the array of the NS is significantly improved without aggregations and exhibits a filling fraction higher than 85% (Figure 2c). After the PMMA removal, no considerable changes in the array are found, and the Au clusters located on PMMA and outside the array could be completely removed (Figure S10).

## Tunable Emission of the Coupled Emitters with Metallic Nanostructures

**Figure 3:**
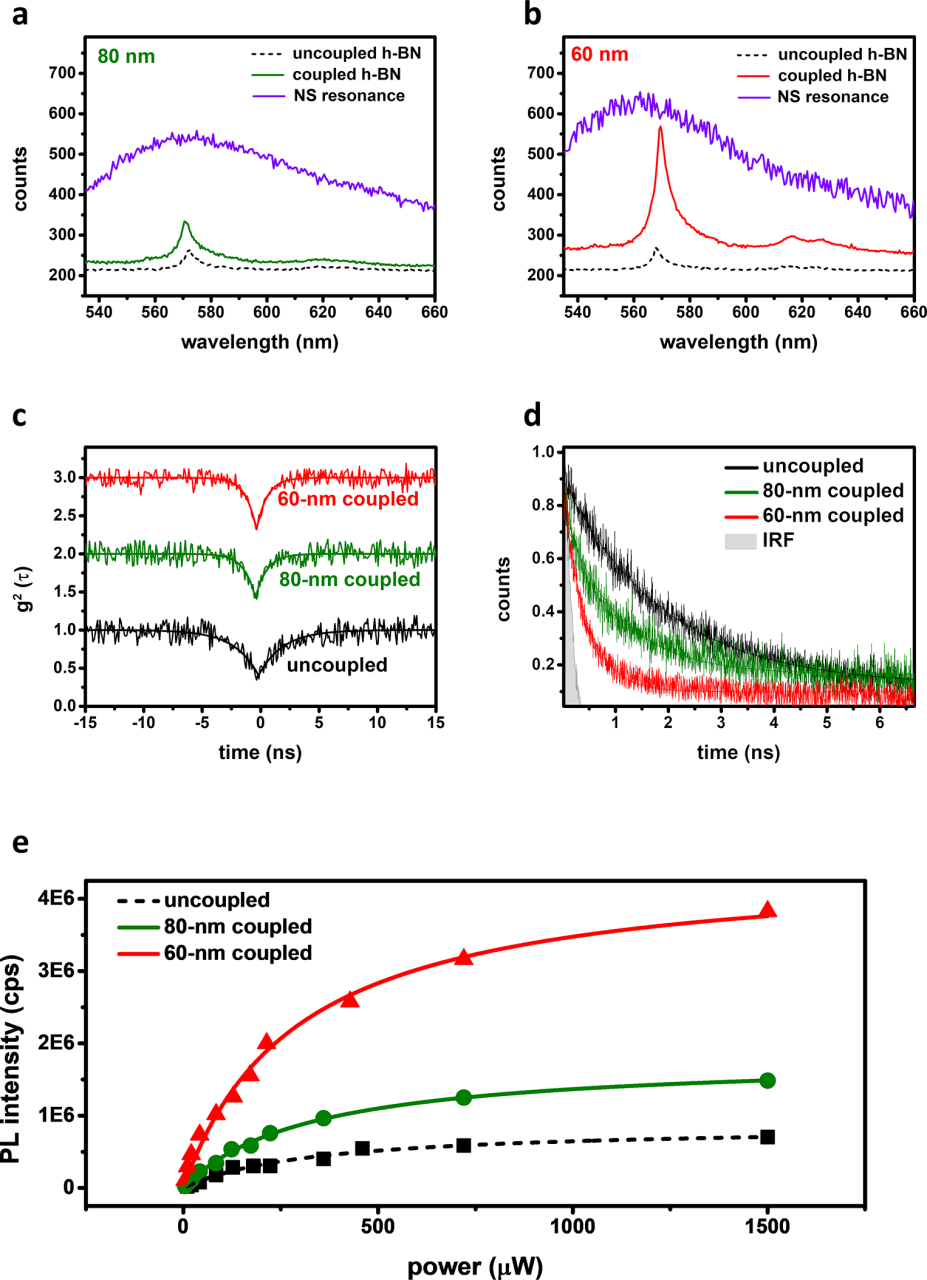
Tunable emission of the coupled emitters with metallic nanostructures. (a) Scattering spectra of the 80 nm NS with LSPR resonant at 573 nm (violet curve). PL spectrum of the uncoupled (black curve) and the coupled (olive curve) SPE. (b) Scattering spectra of the 60 nm NS with LSPR at 560 nm (violet curve). PL spectrum of the uncoupled (black curve) and the coupled (red curve) SPE. (c) A comparison of second-order correlation functions among the emitters located at the 80 nm NS, the 60 nm NS, and outside the NS. (d) Time-resolved PL measurements and (e) saturated fluorescence curves for the emitters located at the three representative locations.

To study the tunable SPE with the array of the metallic single crystals, the size (80 and 60 nm), the shape (isotropic sphere), and spatial distribution (fixed interparticle spacing) of the assembled NS are controlled to study plasmonic interactions among the emitters and the NS. Figure 3a shows scattering spectra from the 80 nm NS (violet) with the plasmonic resonance around 573 nm. [More information on localized surface plasmon resonance (LSPR) can be found in Figure S11]. The Lorentzian fitting of the optical scattering spectra yields a full-width-half-maximum (fwhm) of 0.47 eV, which corresponds to a *Q*-factor of ∼4.6. The typical PL spectra of both the coupled (olive) and uncoupled (black) h-BN emitters exhibit the ZPL at ∼570 nm and a phonon sideband at ∼620 nm. The slight shift of center SPE wavelength could be attributed to nonavoidable variations in the h-BN emitters. Under the same excitation laser power (80 μW), the average ZPL amplitude coupled to the 80 nm NS array is about 95 counts fitted with the Lorentzian function, while the average amplitude of the SPEs uncoupled to the plasmonic array is about 50 counts, and the overall far-field luminous intensity is increased by 200%. In Figure 3b, the scattering spectra of the 60 nm NS show a spectral peak at around 560 nm with the fwhm of ∼0.41 eV corresponding to a larger *Q*-factor of ∼5.4. Note that a considerable enhancement of the emission by ∼600% is observed at the ZPL, while the enhancement at the phonon sideband is ∼200%. Figure 3c shows a second-order correlation function, *g*^2^(τ) from the emitters located at the sites of the 80 nm NS, the 60 nm NS, and outside the assembled NS. The data are fit using a three-level model:^13,40^
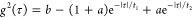
1where *t*_1_ and *t*_2_ are lifetimes of the excited and metastable states, and *a* and *b* are fitting parameters. We used the same model to fit all correlation measurements in this work and obtained the values of *t*_1u_= 1.8 ns, *t*_1–80 nm_ = 0.97 ns, and *t*_1–60 nm_ = 0.85 ns for the emitters at the three representative sites. At the site of the metallic NS, a significant reduction of *t*_1_ by a factor of 2 is due to the plasmonic effect, which is consistent with the reported studies based on the exfoliated samples.^17,19^ However, the reduction in lifetime is not evident in the *g*^2^(τ) functions between the 80 nm NS and the 60 nm NS coupled emitters, which might be due to the very short lifetimes and the finite time response of the system jitter.

For the emitters near the assembled NS, there are two processes contributing to PL enhancement and lifetime reduction. First, the formation of surface plasmons induces an enhancement of the local electromagnetic field, thereby increasing the rate of excitation of the emitters. Second, the emission of the emitters is also intensified because of the enhanced local density of states (LDOS), thereby resulting in a greater probability of transition from the excited to the ground energy state and, hence, leading to an increase in the rate of spontaneous emission by a Purcell factor Fp.^33,43^ The Purcell factor can be expressed as the ratio of the quantum emitter transition rate (Γ) with the NS to native quantum emitter transition rate (Γ_0_) or as the inverse ratio of the lifetime of the quantum emitter (τ) with the NS to native emitter lifetime (τ_0_). Here, the transition rate is the sum of radiative and nonradiative transition rates.
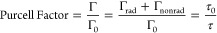
2

To derive a Purcell factor for the coupled system, lifetime measurements of the emitters were recorded using a pulsed laser with 532 nm and a repetition rate of 78 MHz.^24,44^Figure 3d shows the normalized decay curves of three emitters located on the NS and off NS. By using a single exponential fitting function convoluted with the instrumental response function (IRF), we obtain the values of τ_0_ = 2.2 ns, τ_80 nm_ = 1.1 ns, and τ_60 nm_ = 0.45 ns for the uncoupled, 80 nm NS, and 60 nm NS coupled emitters, respectively. The PL intensity increase is in good agreement with the lifetime reduction for the emitter, which suggests an accelerated spontaneous emission rate, with a Purcell enhancement of 4.88 for emitters efficiently coupled to 60 nm NS. To further investigate the PL enhancement, the power-dependent PL measurement was performed. The PL spectrum of SPEs excited with various laser powers are shown in Figure S12, and the photon count curves extracted by the avalanche photodiode versus laser power are shown in Figure 3e. The power-dependent curves are fitted with the following equation:^13−15,19^
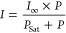
3where *I*_∞_ is the saturated count rate, and *P*_Sat_ is the saturation power. The saturated count rates are 1.8× 10^6^ and 4.5 × 10^6^ counts/s for the 80 nm NS and the 60 nm NS arrangements, respectively. These translate to overall radiative enhancement factors of ∼210% and ∼520% relative to the uncoupled h-BN emitters, which exhibit a saturated intensity of 0.86 × 10^6^ counts/s.^45^ In the saturation regime, the emission is proportional to the radiative lifetime. The factor of enhanced saturation count rate is comparable with the Purcell factor measured in lifetime reduction, which indicates a negligible nonradiative decay channel of the coupled emitters with the single-crystalline plasmonic structure. In the regime before saturation, the emission is proportional to the excitation rate. A steeper slope appears in the coupled SPE, and their saturation powers decrease with the size of the assembled NS, which is consistent with reported papers with the highest enhanced excitation field at the size of 50 nm.^46^ These results confirm efficient localized electric field enhancement and reduced nonradiative scattering of the coupled emitters by the smaller single-crystalline NS.

## Artificial Plasmonic Array of the Single-Crystal Units

**Figure 4:**
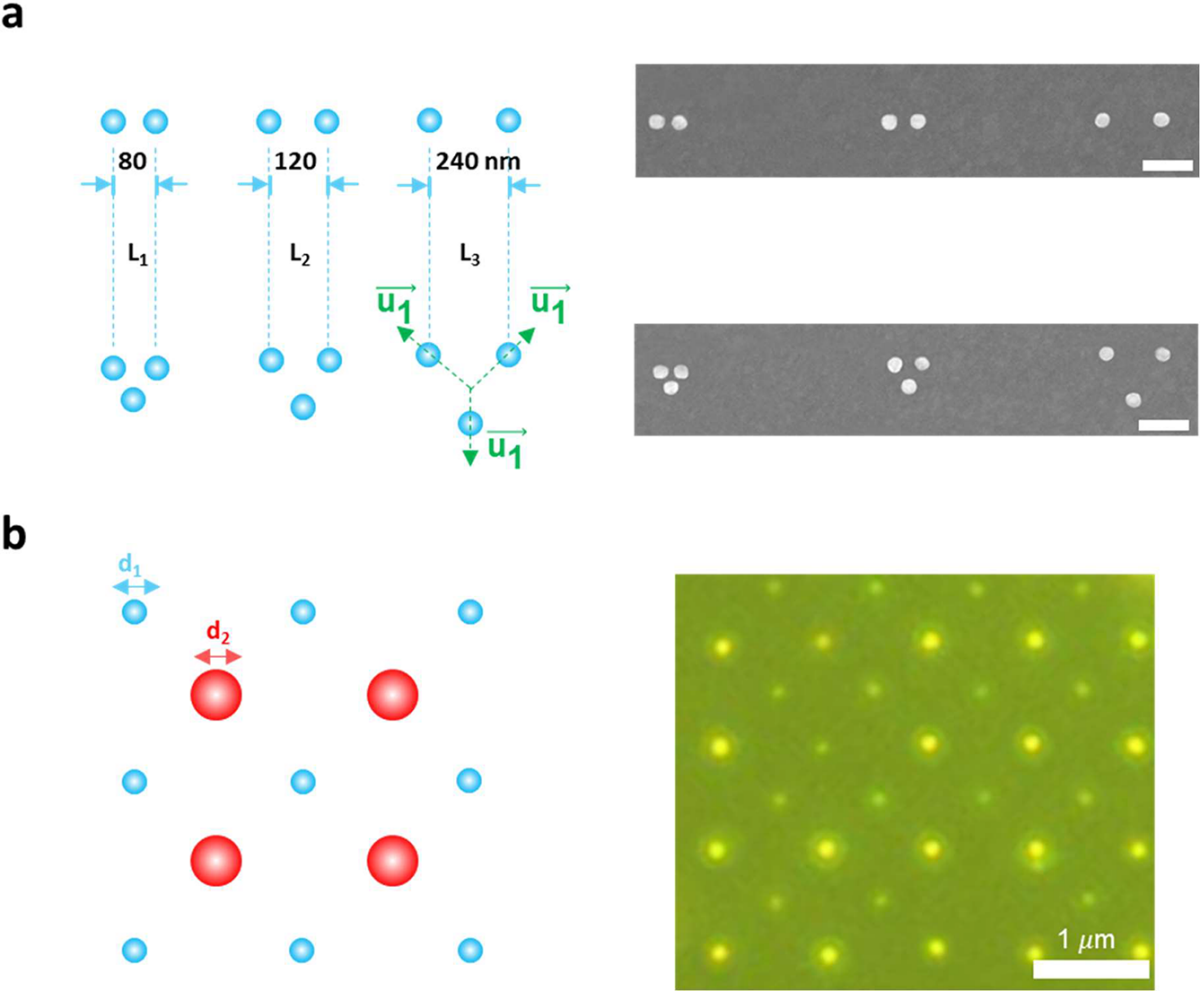
Artificial patterns of the plasmonic array. (a) Designed patterns and SEM images of the dimers and the trimers with tunable interparticle distances (*L* = 80, 120, and 240 nm) and orientations (scale bar: 200 nm). (b) Designed patterns and dark-field microscopy images of the biparticle array with size control (representative size *d* = 65 and 85 nm). Only slight distortions of the assembled units can be observed, which do not affect the effects and observations in this work.

To illustrate the tunability of the fabricated patterns, the dimers and the trimers of the artificially assembled single-crystal units (Au-NS) with various interparticle spacing (*L* = 80, 120, and 240 nm) at specific orientations (*u*_*i*_, unit vectors in the real space) are presented, as shown in Figure 4a. When the hole-to-hole distance equals the size of the NS, the pattern merges together. The force configurations among the nanosized metals would determine the aggregation and assembling of the suspended NS. Artificial patterns for the dimers and the trimers with tunable interparticle distances are clearly demonstrated, thereby indicating a robust control for the assembling process in the EPD reactions (Figure 4a). In addition, the recent theoretical investigation found that when the different sizes of nanoparticles are arranged into bipartite nanoparticle arrays, depending on the relative position of the two particles within the unit cell, these arrays can support lattice resonances with a super- or subradiant character.^47^

In Figure 4b, the array of size-selective bipartite single crystals with a size of sub-100 nm is achieved with the assembly process in EPD reactions. The sequential EPD process consists of two steps. First, the 80 nm NS is assembled into the fabricated nanoholes (85 nm). After the first EPD process, the array of the larger NS is formed, and the smaller holes remain empty. The sample with the preassembled array of the large NS is submerged into the 60 nm NS suspension. With this technique, we can achieve the sorted assembly of bipartite arrays with a complete separation of particles on the basis of their size. By combining the electron beam lithography (EBL)-fabricated nanoholes patterns and controlled synthesis of the single-crystal units, controllable local electric field and its spatial distribution for tunable SPE of the h-BN emitters at RT are achieved. (More information on tuning LSPR can be found in Figure S13). It promises to serve as a robust spectral and spatial filter to improve SPE generation efficiency and indistinguishability deterministically with the Purcell effect.

## Conclusion

We present the wafer-scale fabrication of artificial patterns of single-crystalline NS arrays for deterministic SPE in h-BN at RT. SPE from defective h-BN is enhanced and tuned by the artificial array. An enhancement of the SPE intensity of ∼500% is observed with a radiative quantum efficiency of up to 20% and a saturated count rate in excess of 4.5 × 10^6^ counts/s. The single-crystal nature of the plasmonic units significantly enhances localized electric fields and reduces the nonradiative scattering of coupled emitters. Tunable emissions of synthetic vdW materials with artificial plasmonic devices move a significant step toward quantum information and nanophotonics.

## Data Availability

The data that support the findings of this study are available within the paper and the Supporting Information. Other relevant data are available from the corresponding authors upon reasonable request.

## Supporting Information Available

Description of the material, details of the EPD reactions, and experimental details, as well as growth process, SEM, PL, Raman, and correlation function data (PDF)
