# Supplementary material for: Tunable Single-Photon Emission with Wafer-Scale Plasmonic Array
Source: Nano Lett. 2024 Feb 15;24(11):3395–403. doi: 10.1021/acs.nanolett.3c05155 (PMC10958497; doi:10.1021/acs.nanolett.3c05155)
Supplement: Supplementary file 1 — nl3c05155_si_001.pdf [file nl3c05155_si_001.pdf]

Supporting Information

Tunable Single-Photon Emission with Wafer-scale Plasmonic Array

Chun-An Chen[1,4], Po-Han Chen[1,4], Yu-Xiang Zheng[1,4], Chiao-Han Chen[1], Mong-Kai Hsu[1], Kai-Chieh Hsu[1], Ying-Yu Lai[12], Chih-Sung Chuu[3], Hui Deng[2] and Yi-Hsien Lee[1*]

[1]Department of Materials Science and Engineering, National Tsing Hua University, Hsinchu 30013, Taiwan

[2]Department of Physics, University of Michigan, Ann Arbor, MI, 48109-2122, USA

[3]Department of Physics, National Tsing Hua University, Hsinchu 30013, Taiwan

[4]These authors contribute equally to this work: Chun-An Chen, Po-Han Chen, Yu-Xiang Zheng

*E-mail: yhlee.mse@mx.nthu.edu.tw

Summary

S1. Method

S2. Enlarged SEM image of isolated nanosphere.

S3. Controlled synthesis of the units for tunable localized surface plasma resonance (LSPR)

S4. Preparation of high-density quantum emitters of scalable h-BN

S5. Characterization of h-BN after transfer

S6. Angle-resolved polarization SPE of the h-BN emitter

S7. Comparison of the fabricated array of single-crystalline and polycrystalline units (EPD and E-gun evaporation)

S8. Additional measured points of the second-order correlation function

S9. Voltage-dependent EPD reactions for the array of the assembled units

S10. Cycling-dependent EPD reactions for filling fraction of the fabricated array

S11. Optical image of gold NS deposition before and after removal of PMMA.

S12. Variations of resonance spectra of AuNS in different measurement conditions:

method, sample type, and environmental issues

S13. Saturated emission and power-dependent PL of the h-BN emitters

S14. Synthesis of the single-crystal units with tunable shapes for resonance control

S15. Setup and details of optical measurements.

S16. Reference

S1. Methods

Particle assembly: The conducting layers of the Au (30 nm) / Ti (2 nm), deposited on the sapphire substrate by an e-gun evaporator, enable the following e-beam lithography (EBL) for nano-fabrication and work as the electrode for the EPD reactions. A high resolution of 10-20 nm in the EBL-based nano-fabrication enables precise control of the size, shape, and spatial distribution of the designed holes in the photoresist (PR) layer. A positive PR layer of the PMMA (950 K A4, Microchem Inc.) was spin-coated on the Au/Ti-covered sapphire at 5000 rpm for 40 s, and further baked at 150 °C for 5 min to form template film. Then, the PMMA-coated samples were exposed to e-beam (ELS-7800, Elionix Inc.) and immersed in the solutions of MIBK and IPA for the development process. Isotropic and spherical patterns in the template film were designed to fit the size of the NS. The hole diameter is ~5% larger than the NS diameter for better filling of the assembled units. The single-crystal NS suspended in the solution was prepared with tunable sizes, and shapes using seed-mediated synthesis.[1-3] The assembled NS was filled into the patterned holes in the EPD reactions with an optimized NaCl concentration (0.5 mM) and a time of 60 s for each cycle. The variable-bias EPD reactions were performed by applying gradually-increased voltages from 3.5 to 3.7 V for a higher filling fraction.

Preparation of h-BN and integration: The h-BN layer studied here was grown by LPCVD on a Cu foil (0.025 mm, 99.8%, Alfa Aesar) at 1050 ℃ using ammonia borane (BH_3NH_3, Sigma-Aldrich). The h-BN layer was transferred to the SiO_2/Si wafer for pre-characterization by water-soluble polyvinyl alcohol (PVA) assisted transfer. For the fabrication of the PVA film, an aqueous solution was prepared by mixing PVA (5 g), glycerin (1 g), and ultra-pure deionized water (100 ml, 18.2 $M\Omega$). The PVA solution was drop-cast on top of the as-grown h-BN on the Cu foil. After the drop-casting, the PVA-covered h-BN/Cu foil sample was heated at 80 °C to ensure complete drying. The PVA-covered h-BN film was peeled off from the Cu foil and transferred onto the SiO_2/Si substrate for checking the emitter density. The

PVA-covered h-BN samples were soaked in the hot DI water (80 °C) for 4 hours to assure complete removal of the PVA. Prior to further integration, PL mapping of the synthesized h-BN was performed to confirm a high density of uniformly-distributed quantum emitters in the h-BN. After the confirmation of the emitter density, the h-BN was removed from SiO_2 and further transferred to integrate with the NS array using the PMMA-assisted transfer.

Optical measurement: All optical measurements reported here were performed via a home-built confocal microscope with an objective lens with a numerical aperture of NA = 0.9. All optical measurements were performed at room temperature. PL spectra were acquired with an Andor spectrometer with a measured spectral resolution of 0.3 nm and a cooled charge-coupled camera. A free-space Hanbury Brown and Twist interferometer was utilized for characterizing the second-order photon correlation function. The photon counting was performed using two avalanche photodiodes (APDs). No filters or background corrections were utilized to spectrally select one quantum emitter. Time-resolved PL measurements were performed with a supercontiuum pulsed laser (NKT SuperK Extreme), with a 5 ps pulse width and a 78 MHz repetition rate and the wavelength of light was set to 532 nm. (Supplementary Fig.14).

S2. Enlarged SEM image of isolated nanosphere.

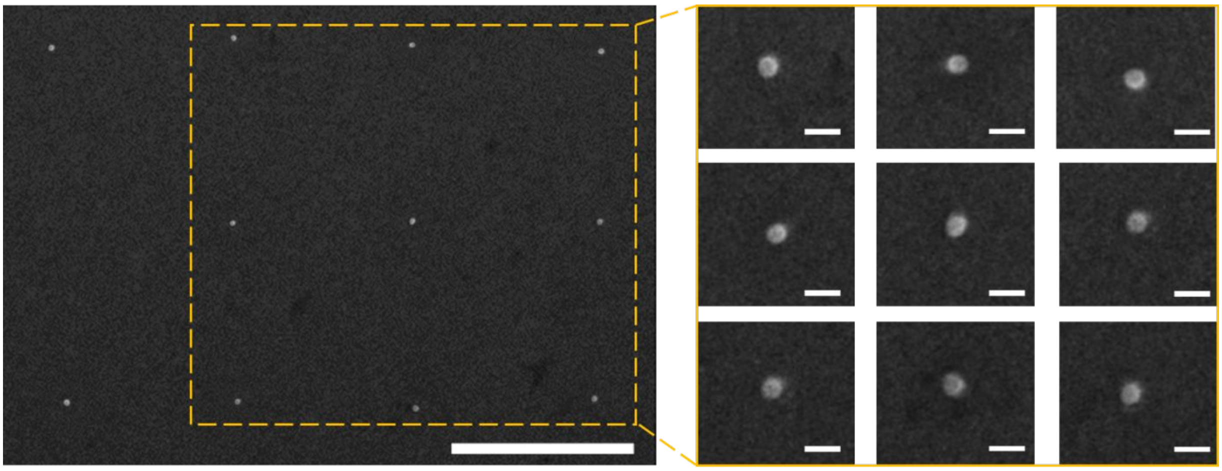

Figure S1. A magnified SEM image of the surface morphology of gold nanoparticles exhibiting the distinct morphology of isolated gold nanoparticles. (Scale bar: 2 μm and 100 nm)

S3. Controlled synthesis of the units for tunable localized surface plasma resonance (LSPR)

Single crystals of the gold nanospheres (Au-NS) were synthesized by the seed-mediated growth with the precursors of Gold (III) chloride trihydrate ($HAuCl_4 \cdot 3H_2O$, ≥ 99.99 %, Sigma-Aldrich), ascorbic acid (AA, ≥98%, Sigma-Aldrich), sodium borohydride ($NaBH_4$, 99%, Sigma-Aldrich), cetyltrimethylammonium bromide (CTAB, ≥98%, Sigma-Aldrich), and cetyltrimethylammonium chloride (CTAC, 96% in water, Sigma-Aldrich). The suspended Au ions in the solution (Au^+) are prepared by rapid reduction reactions of the $HAuCl_4$ (5 ml, 0.25 mM) by a strong reducing agent $NaBH_4$ (600 μL, 10 mM). The Au^+-rich solution was then added to the concentrated CTAB solution (5 ml, 100 mM). The CTAB molecule-covered gold clusters are the seeds for controlled synthesis of the gold nanostructures with tunable shapes and sizes in multiple stages. The initially-nucleated single crystals of the seeds exhibit a diameter of ~9 nm and the gold NS are prepared in the following growth reactions for the isotropic shape with sizes ranged from 9 to 104 nm. (Supplementary Fig. 2a)

The size of the synthesized units is tuned by controlling the seed amounts in successive growth (Supplementary Fig. 2b). With reduced amounts of the nucleation sites, more suspended gold ions in the solutions will attach to the seeds for larger sizes of the synthesized single-crystal units. With the increased sizes of the as-prepared units, the wavelength of the absorption peak is red-shifted to longer wavelengths as shown in Supplementary Fig. 2c. Supplementary Fig. 2e demonstrates tunable absorption by correlating spectra of the characteristic wavelength with the size of the synthesized units.

With the increased size, the observation of a clearly broadened bandwidth of LSPR indicates retardation effect of the plasmon (Supplementary Fig. 2d),[4] which is consistent with the previous report. Compared with the non-isotropic counterparts, a spherical and single-crystal unit with a nanoscale size is ideal for an understanding of fundamental issues with simplified reactions, models, and computational simulations. Spherical nanostructures enable precise SPR spectra to exclude undesirable SPR signals at longer wavelengths originating from the corner and various facets.[5] With the above considerations, the spherical Au single crystals are selected as building blocks for the construction of the designed pattern. Mass production of the single-crystal units is achieved and the fabricated array demonstrates a high uniformity of the assembled units on the size and shape, which move a significant step toward nano-photonics and quantum optics.

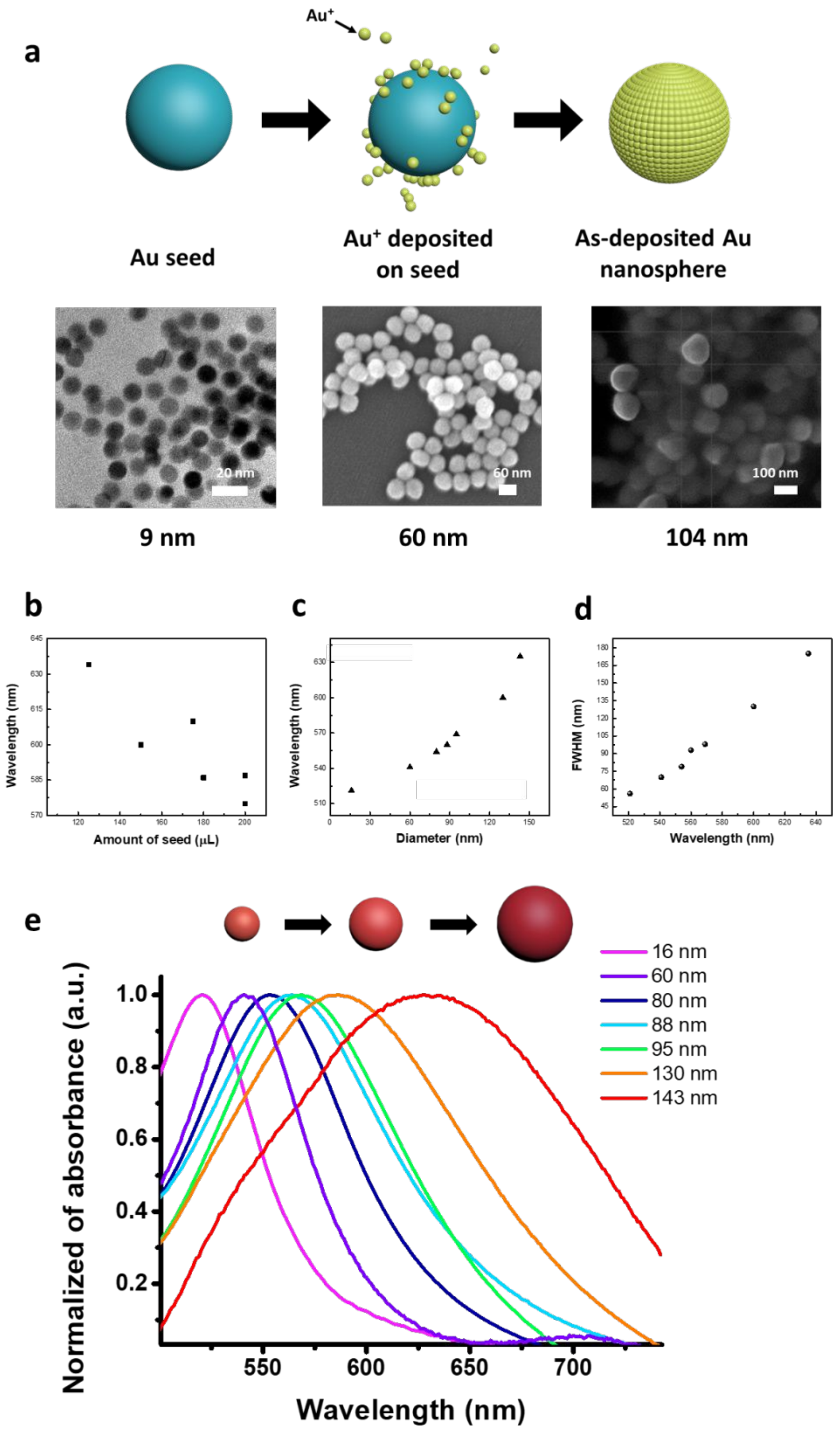

Supplementary Figure 2. Controlled synthesis of the single-crystal units for tunable LSPR.

a) Schematic diagram of seed crystal growth and SEM image of single-crystal NS with tunable diameters from 9 to 104 nm. b) Plot of the wavelength of LSPR as a function of the amounts of seeds. With reduced amounts of the nucleation sites, more suspended gold ions in the solutions will attach to the seeds and form larger sizes of the synthesized single-crystal units. c) Size-dependent wavelength of LSPR of the gold NS. As the size increases, the wavelength of LSPR

red-shifts. d) Size-dependent FWHM of the gold NS. As the size increases the full width at half maximum (FWHM) gradually increases. e) Absorption spectrum of tunable sizes of the NS.

S4. Preparation of high-density quantum emitters of scalable h-BN

The defective h-BN layer was grown on the Cu foils (0.025 mm, 99.8%, Alfa Aesar) at 1050 °C using ammonia borane (BH_3NH_3, Sigma-Aldrich) by low-pressure chemical vapor deposition (LPCVD), which is similar to the reported paper [6]. The synthesized h-BN layer was transferred to the SiO_2/Si wafer with the polyvinyl alcohol (PVA)-assisted transfer (Supplementary Fig. 3b). The PVA-covered samples are soaked in the D.I. water at 80 °C to assure no detectable polymer residues (Supplementary Fig. 3c). The OM image clearly demonstrates a large area and uniform layer of the synthesized h-BN. Prior to the integration with the metallic units, the emission of the defective h-BN was examed to confirm a high density of quantum emitters in the as-grown h-BN. Supplementary Fig. 3e is a $60 \times 60\ \mu m^2$ confocal map of the sample with uniform emission. A fine scan of $5 \times 5\ \mu m^2$ presents a blurry image of the uniformly-distributed emitters due to resolution limit of the laser.

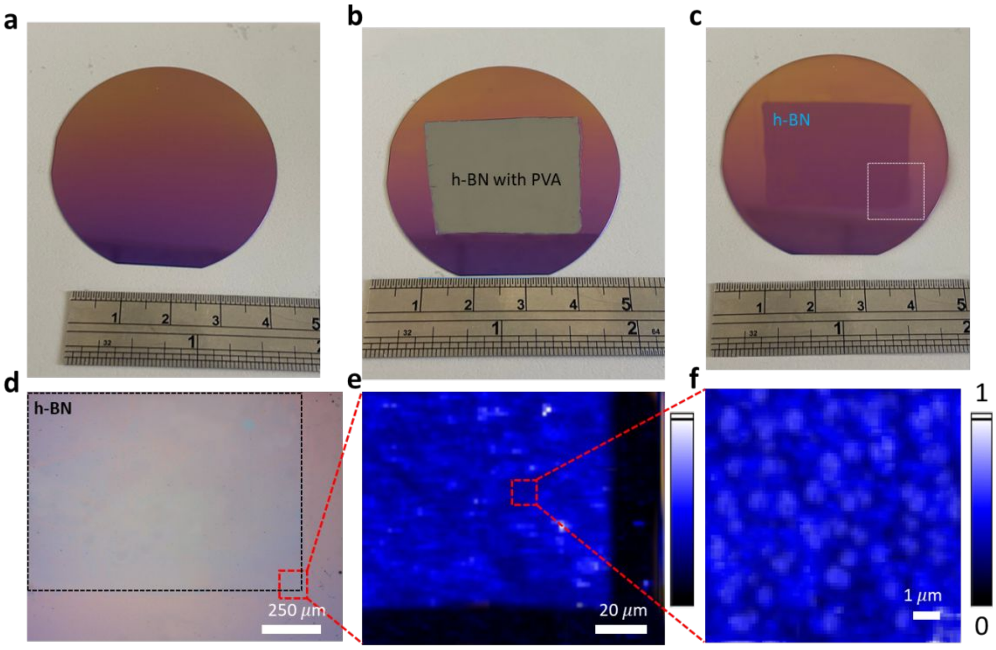

Supplementary Figure 3. Preparation of high-density quantum emitters of the h-BN.

a) A 2-inch SiO_2/Si wafer substrate for checking of emitter density. b) The PVA-covered h-BN is transferred from the copper foil (3×2 cm) onto the wafer. c) The transferred h-BN on SiO_2/Si wafer after removal of the PVA. d) OM image of the h-BN in the marked (white rectangular) corner. e) PL maps of the defect emission. f) Fine scan of the PL maps of the emitters in the h-BN.

S5. Characterization of h-BN after transfer

Thickness of the synthesized h-BN is confirmed using atomic force microscopy (AFM) in the area with clear edges of h-BN and substrates as shown in Supplementary Fig. 4c and 4d. The average height of the synthesis h-BN is 1.94 nm with an average roughness (Ra) of 0.4 nm. A flat, uniform, and clean surface with reduced surface roughness is observed. Supplementary Fig. 4e illustrates the representative Raman spectra obtained at different locations. Compared to the peak at 1367cm^{-1} for vibration modes of E_{2g} mode of the monolayer h-BN, the peak shows a blueshift and appears at 1365 cm^{-1} because of its increased thickness. [7] Our synthetic h-BN exhibits a Raman peak at ~1366 cm^{-1} with FWHM values spanning from 19.1 to 24.0 cm^{-1}.

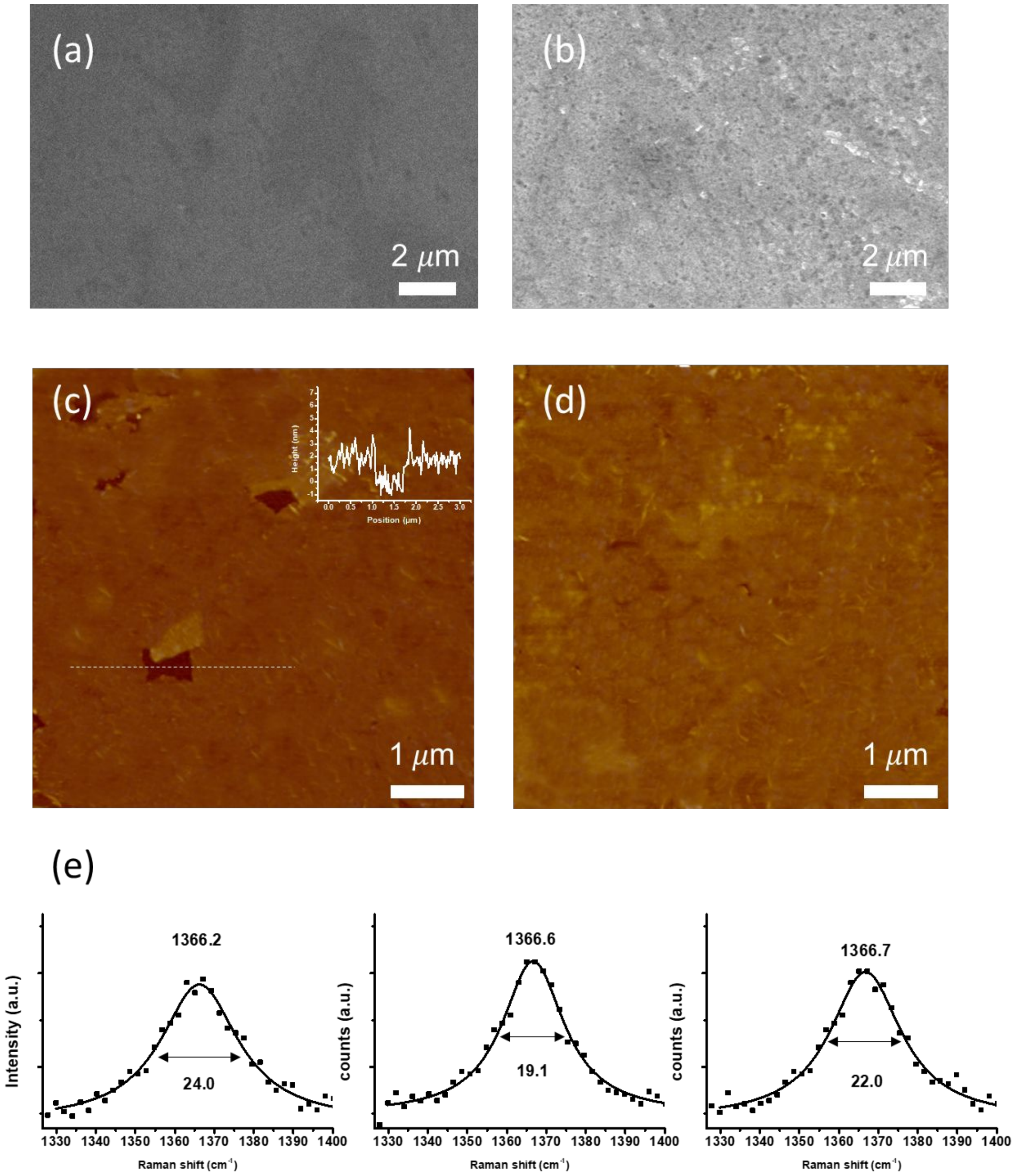

Supplementary Figure 4. Characterization of h-BN after transfer.

a) and b) SEM image of as-grown h-BN on copper foil. c) and d) AFM image of the transferred h-BN on SiO2/Si wafer after removal of the PVA. Height profile of the line scan of the h-BN over white dash line. e) Representative Raman spectra obtained at different locations (in Supplementary Fig. 3).

S6. Angle-resolved polarization curve from SPE of the h-BN emitter

Degree of polarization (DOP) for the h-BN emitters, calculated using the formula

$\frac{I_{max} - I_{min}}{I_{max} + I_{min}}$, displays a range from 0.8 to 0.9 and this value is comparable to the reported record. [7] No detectable changes in the polarization direction prior to and subsequent to the coupling process.

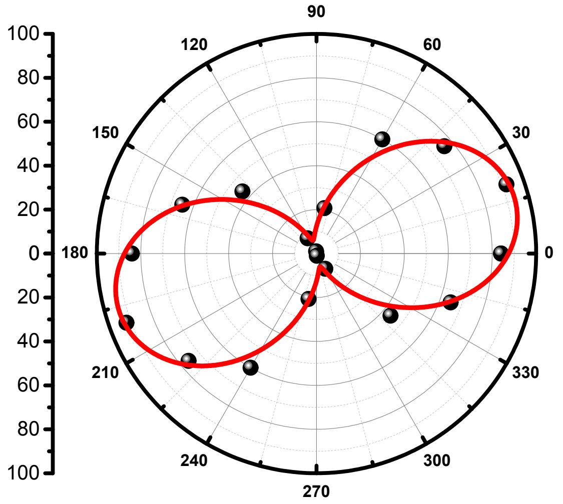

Supplementary Figure 5. Angle-resolved polarization curve from SPE of the h-BN emitter.

S7. Comparison of the fabricated array of single-crystalline and polycrystalline units (EPD and E-gun evaporation)

Fabrication of the designed patterns of the assembled nano-unit by EPD (single-crystal NS) and e-gun evaporation deposition (polycrystalline aggregates) are performed and studied. Both processes utilize electron beam lithography to fabricate the designed hole pattern with a diameter of 65 nm. An atomic force microscope (AFM) was performed to evaluate the height, morphology, size, and quality of the assembled units. AFM image of the assembled unit of the colloidal reaction-synthesized gold NS displays single-crystal signatures with uniform morphology and shape (Supplementary Fig. 6a) while that of the e-gun deposited gold exhibits poly-crystalline morphology with considerable boundaries. The line profile of the assembled

single-crystal NS units shows a uniform height of ~60 nm with smooth morphology and an isotropic geometry as shown in Supplementary Fig. 6c. In contrast, the polycrystalline units exhibit two protrusions due to the existence of grain boundaries.

The enhancement from e-beam evaporated polycrystalline units is not observable from multiple coupling sites, and in some cases, it can even be quenched with a large scattering background as shown in Supplementary Fig. 6d. These observations better support the point mentioned that the bottom-up route would be a promising option for (scalable) applications of quantum information.

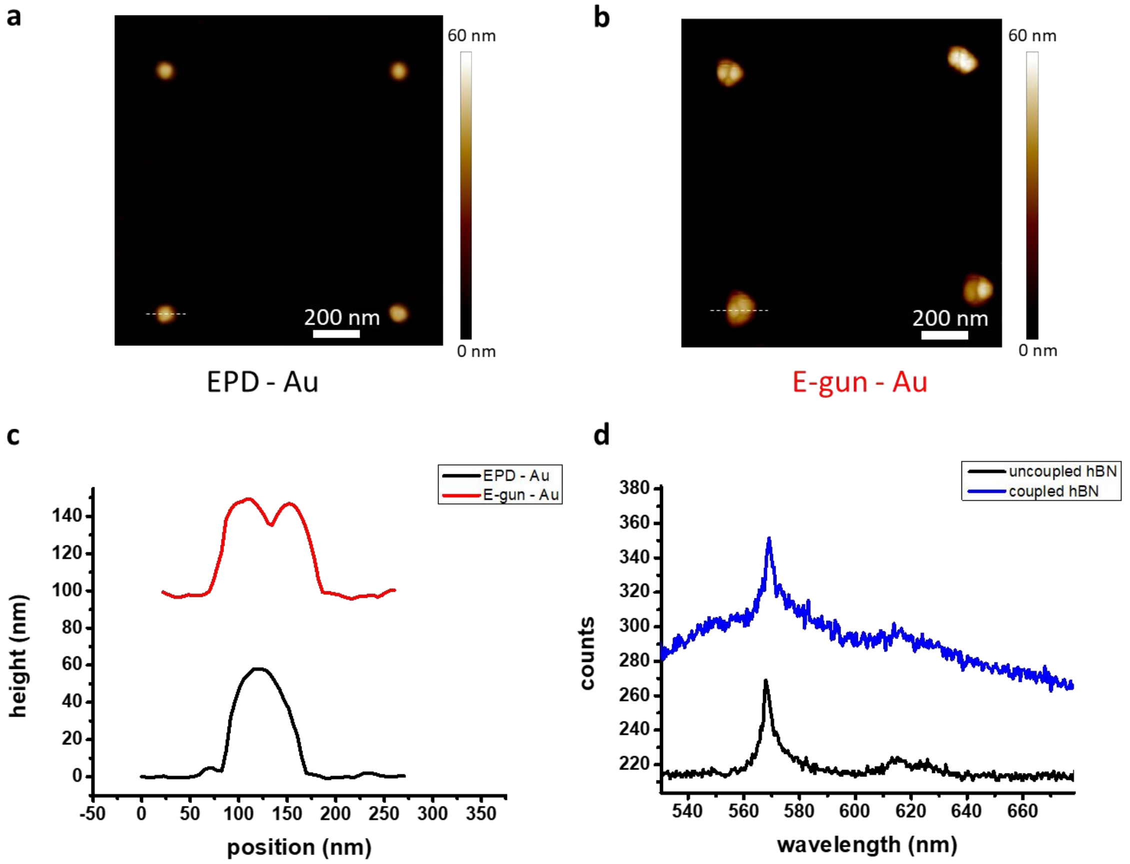

Supplementary Figure 6. Comparison of the fabricated array of single-crystal and polycrystalline units.

AFM images on the designed pattern of the assembled units using a) EPD reactions and b) e-gun evaporation. c) Height profile of the line scan over the white dash lines in figures a) and b)

with black (EPD) and red curves (E-gun). d) PL spectrum of the uncoupled (black curve) and the E-gun-Au coupled (blue curve) SPE.

S8. Additional measured points of the second-order correlation function

Additional experimental data pertaining to the $g^{(2)}$ values of the uncoupled and coupled h-BN emitters are presented in Supplementary Fig. 7 to provide a more comprehensive illustration. It is evident from the results that the $g^{(2)}$ values of the emitters decrease to an average value of approximately 0.32 upon coupling with gold nanoparticles.

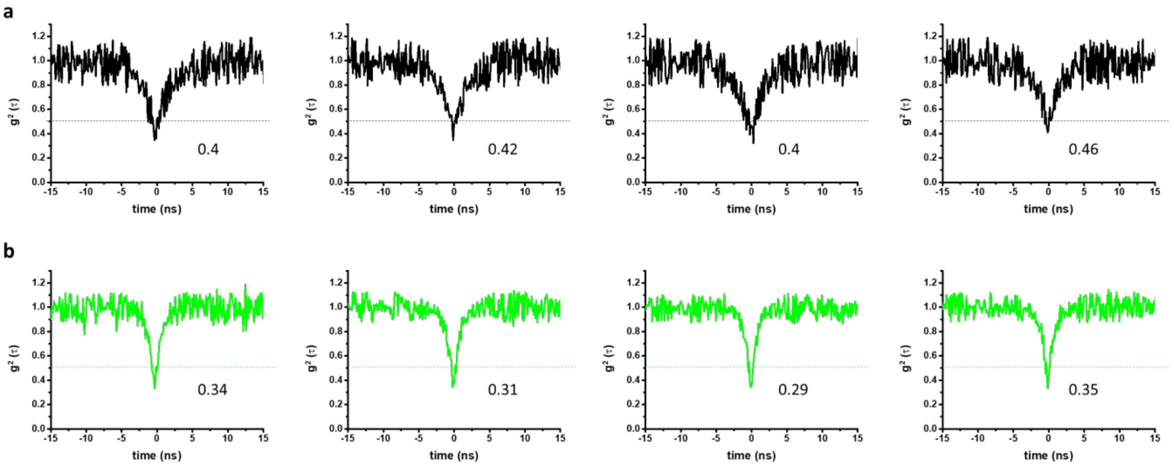

Supplementary Figure 7. Additional measured points of the second-order correlation function. Second-order correlation function for both a) the uncoupled (black curve) and b) coupled (green curve) SPE.

S9. Voltage-dependent EPD reactions for the array of the assembled units

The filling fraction of the designed patterns with the assembled NS units in EPD reactions is significantly determined by the applied voltages and the NS size. For the optimized conditions in the EPD reactions for a high filling fraction with monomer, voltage-dependent EPD reactions were performed. Supplementary Fig. 8b shows magnified images of the designed patterns of the assembled 60-nm NS. In the constant-voltage EPD reactions, an average filling fraction of 4% was found. In the variable-voltage EPD reactions, a higher fraction of assembled

NS is achieved with increased voltages. In the sequential EPD reactions at 3.7 V, the filling fraction of patterned holes is increased to 58%. With the increase of the electric field in the EPD reactions, more aggregations of the NS are clearly observed.

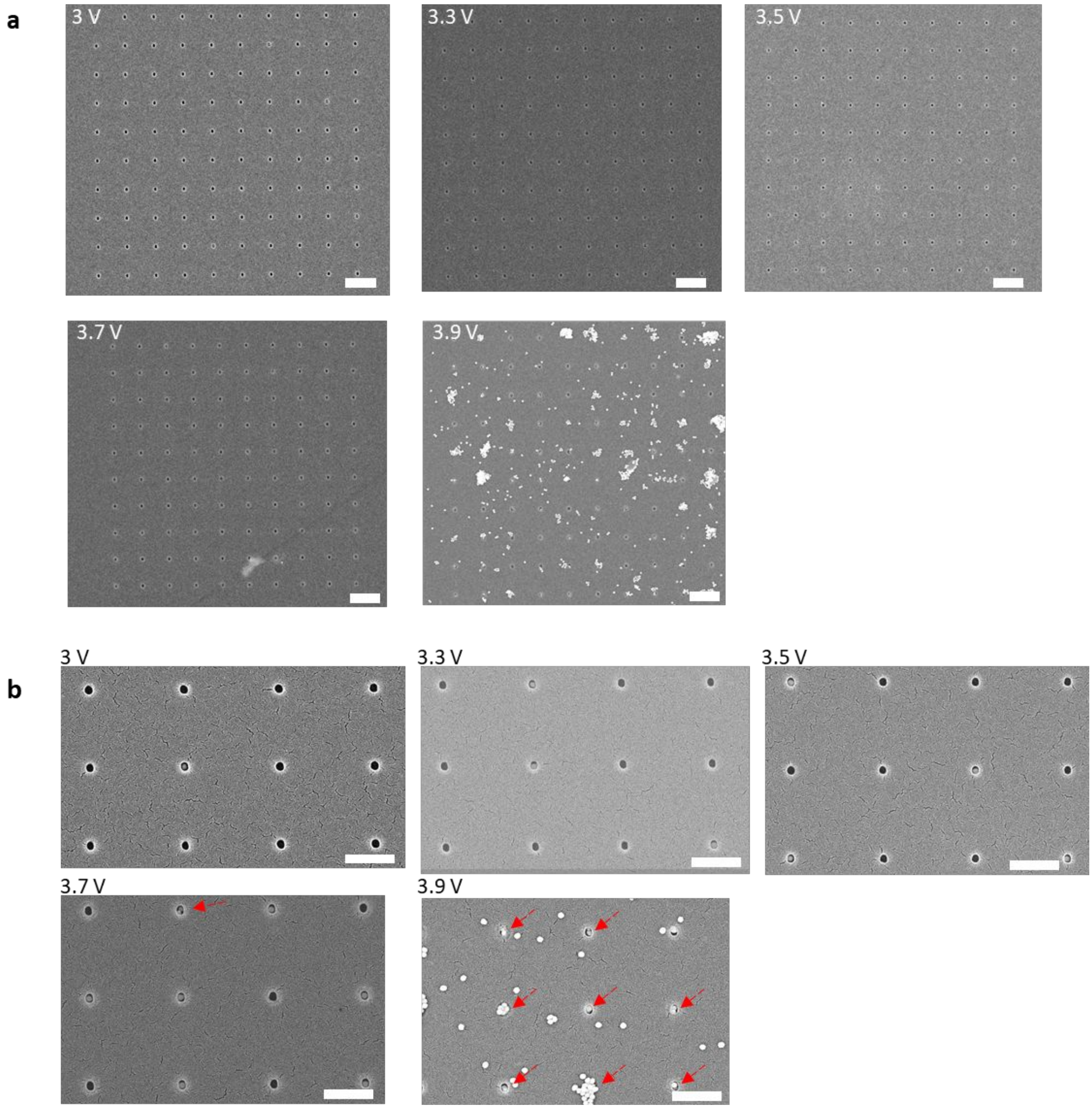

Supplementary Figure 8. Voltage-dependent EPD reactions for filling the assembled units. a) Low magnification SEM image of the assembled NS. (Scale bar: 1 μm) b) Zoom-in SEM image of the assembled NS. The marked red arrows are represented as the regions with the aggregated NS. (Scale bar: 500 nm)

S10. Cycling-dependent EPD reactions for filling fraction of the fabricated array

Supplementary Fig. 9 presents dark field images of the array of assembled NS in the constant-voltage EPD reactions with the three representative voltages: 3V, 3.5 V, and 4 V (breakdown voltage). The EPD reactions are cycling for 7 times at the three representative voltages and each cycle of the reactions is carried out with a fixed operation time of 60 s. The filling fraction of the hole patterns is quantitatively determined using the software of image-J. Each designed array is composed of 16x16 holes. In the constant voltage EPD reactions at 3 V (3.5 V), the filling fraction of the assembled units is increased from 4% (33%) to 21% (78%) with 7 cycles. In the EPD reactions at a voltage of 4V, damage of the PMMA template is commonly observed.

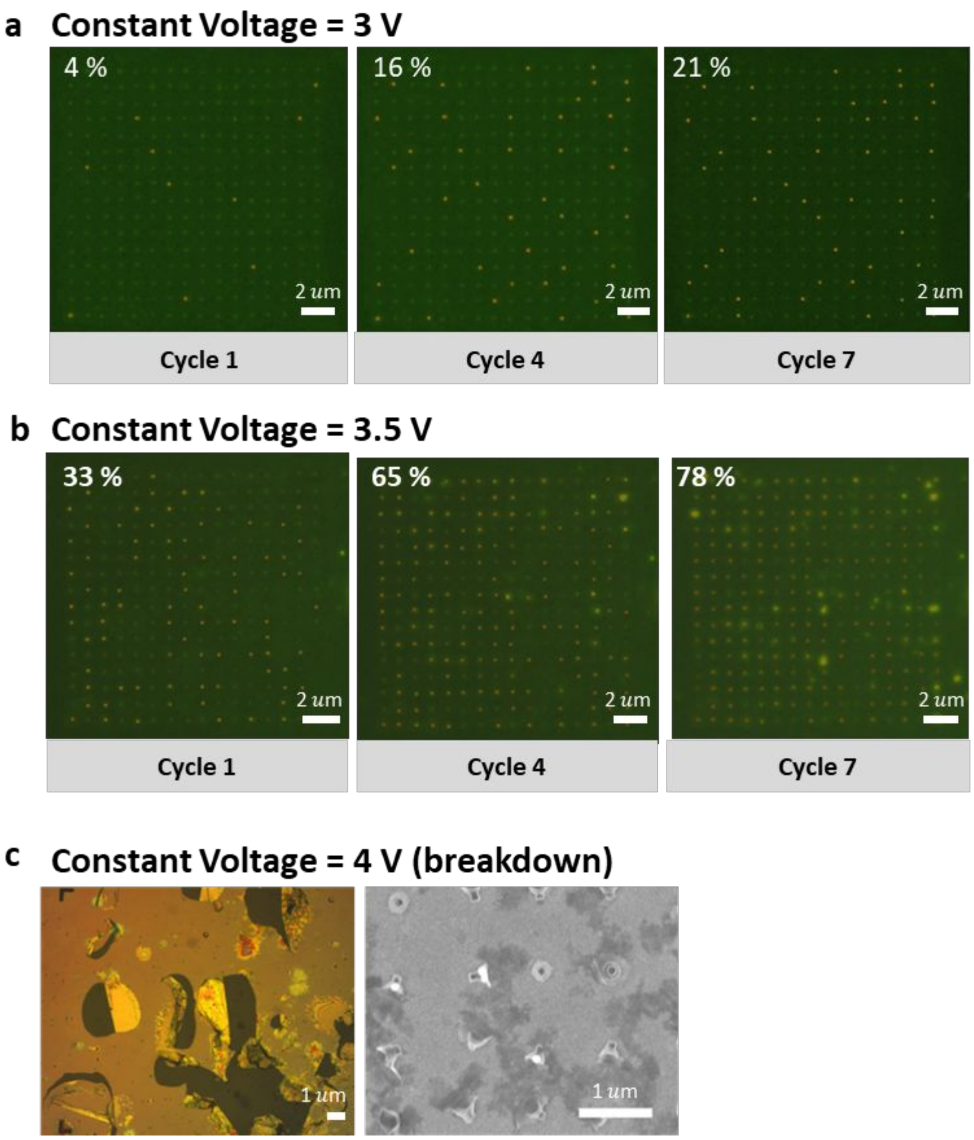

Supplementary Figure 9. Constant-voltage-EPD reactions for artificial patterns of the assembled units.

Dark field image of 16 × 16 holes with 7 cycles of constant-voltage-EPD reactions at a) 3 V and b) 3.5 V c) Bright field OM image shows that PMMA template was peeled off once reaching breakdown voltage (4 V).

S11. Optical image of gold NS deposition before and after removal of PMMA.

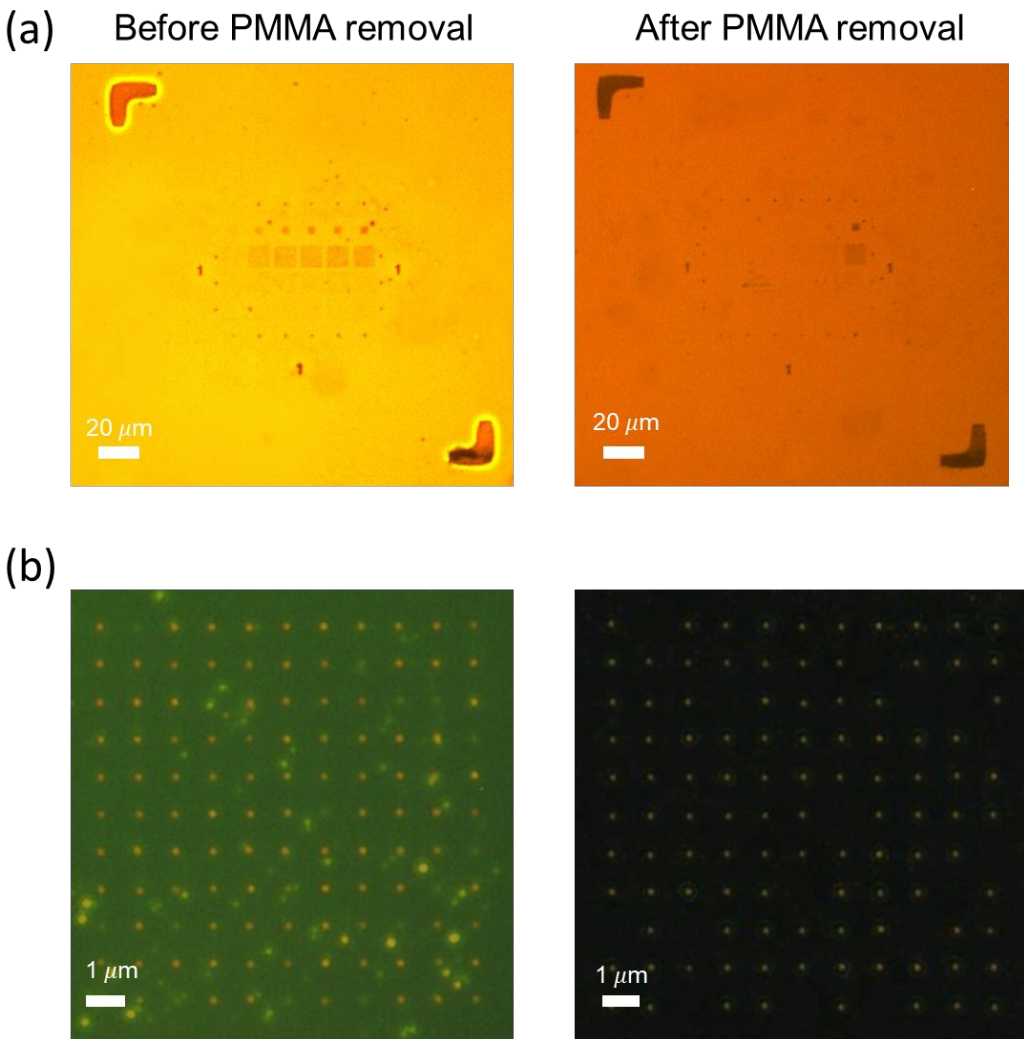

Supplementary Figure 10. The gold NS array before and after PMMA removal.
a) Optical image of the patterned array before and after acetone cleaning. The effectiveness of acetone in removing PMMA is demonstrated by the conspicuous change in color of the marker situated at the corner. b) Dark field image of the patterned array before and after acetone cleaning.

S12. Variations of resonance spectra of AuNS in different measurement conditions: method, sample type, and environmental issues

To explain possible spectral variations on the resonance of the Au (Fig. 3 and Supplementary Fig. 2c), diverse measurement conditions are discussed, including characterization methods, sample type, and environmental issues. A 20 nm spectral shift difference between the scattering spectra and the extinction spectra can be attributed to

variations in the two different characterization methods. Specifically, the extinction spectra were obtained with the measurement at the ensemble level in an aqueous solution through UV-visible spectroscopy as shown in Supplementary Fig. 11a. In contrast, the scattering spectra were acquired with the single particle measurements on the dry samples using a reflectance dark-field microscope as shown in Supplementary Fig. 11b. It is possible to observe a peak shift in the peak positions of the scattering spectra compared to the extinction spectra, which is consistent with the reported literature.[8]

More details are included for the resonance spectrum with extinction spectroscopy (Supplementary Fig. 2). A drop of the solution containing synthesized gold nanospheres is deposited onto a SiO_2 substrate. The size of the nanoparticles is determined through scanning electron microscopy (SEM) images. The resonance spectrum associated with the gold nanoparticles is identified through UV-visible spectroscopy of the aqueous solution. Moreover, more details are also presented for the resonance spectrum with scattering spectroscopy (Fig. 3a and 3b). Differential reflectivity is measured using reflective-type scattering spectroscopy to identify the resonance spectrum resulting from the coupling between the gold nanoparticles, buffer layer (Al_2O_3), and hexagonal boron nitride (h-BN) on Au (30 nm) / Ti (2 nm), which is deposited on the sapphire substrate. The measurement approach is similar to established measurement setups in the literature.[8] The scattering efficiency of gold nanospheres decreases as their size decreases, and the reduced intensity of the white light source at shorter wavelengths may result in increased noise levels.

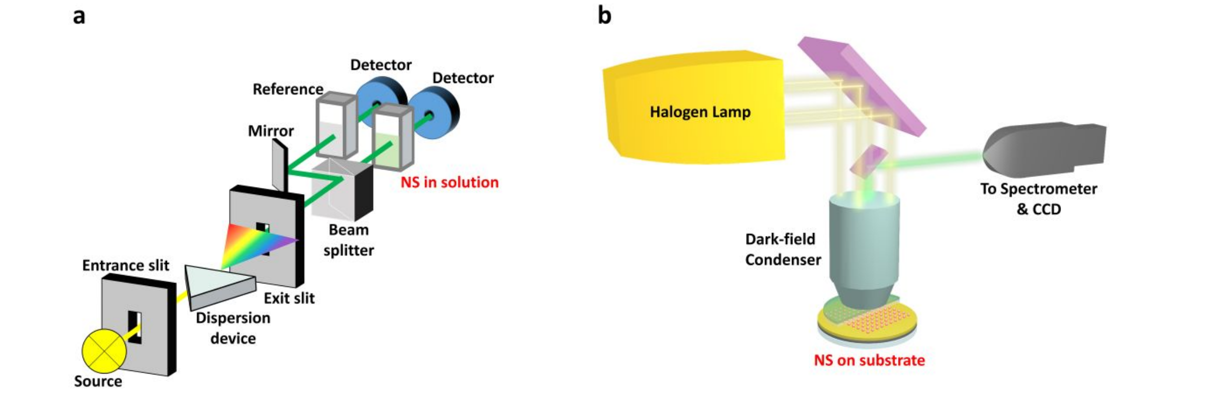

Supplementary Figure 11. Schematic of measurement setups for resonance spectra.

a) UV-Vis measurement for the NS in solution. b) Reflectance dark field microscope for the NS on a substrate.

S13. Saturated emission and power-dependent PL of the h-BN emitters

The photoluminescence (PL) intensity and the photon count rates are simultaneously recorded with the 10/90 beamsplitter as incident power changes. 90% of the photon are directed to the single photon avalanche diode and the photon count rates are extracted with the background correction. Data sets are plotted as a function of corresponding power of incident light for the uncoupled SPE and the coupled SPE at 80-nm NS, and 60-nm NS .

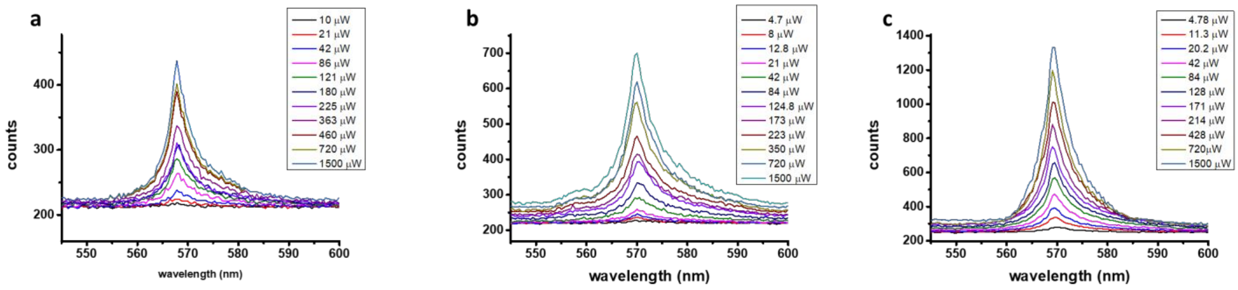

Supplementary Figure 12. Saturated emission and power-dependent PL of the h-BN emitters.

Power-dependent PL measurement of a) the uncoupled SPE, and the coupled SPE at b) 80-nm NS and c) 60-nm NS

S14. Synthesis of the single-crystal units with tunable shapes for resonance control

Variations in defect configurations and spatial distribution of the h-BN emitters resulted in the extraordinarily broad spectral distribution of the zero phonon lines (ZPL). To realize tunable SPE with reduced variations, spectral filtering of the SPE is achieved based on plasmonic resonance by tuning the size and shape of the assembled units in the artificial array. The size of the gold nanospheres is significant for enhanced emission properties of the h-BN SPE. With the increased size of the gold nanospheres, plasmonic resonances shift to higher wavelengths with broader FWHM, resulting in a decreased overlap between the plasmonic field and the spectral emission of the SPE (Supplementary Fig. 2). Moreover, the utilization of smaller gold nanospheres facilitates improved precision in the positioning and coupling of SPEs in relation to the more localized enhanced field.

With the control of size and shape, spectral variations of the ZPL are filtered via field enhancement generated from the assembled units at deterministic sites. Supplementary Fig. 13a presents the synthesis of single-crystal Au nanostructures with tunable shapes and sizes for controllable plasmonic resonance. Supplementary Fig. 13b and 13c demonstrate robust tunability on the resonance wavelength of the synthesized units by tuning various aspect ratios for a controlled geometry. The h-BN emitters are integrated with the array of assembled units with controllable LSPR wavelength, which improves the brightness and indistinguishability of the h-BN emitters with the Purcell effect.

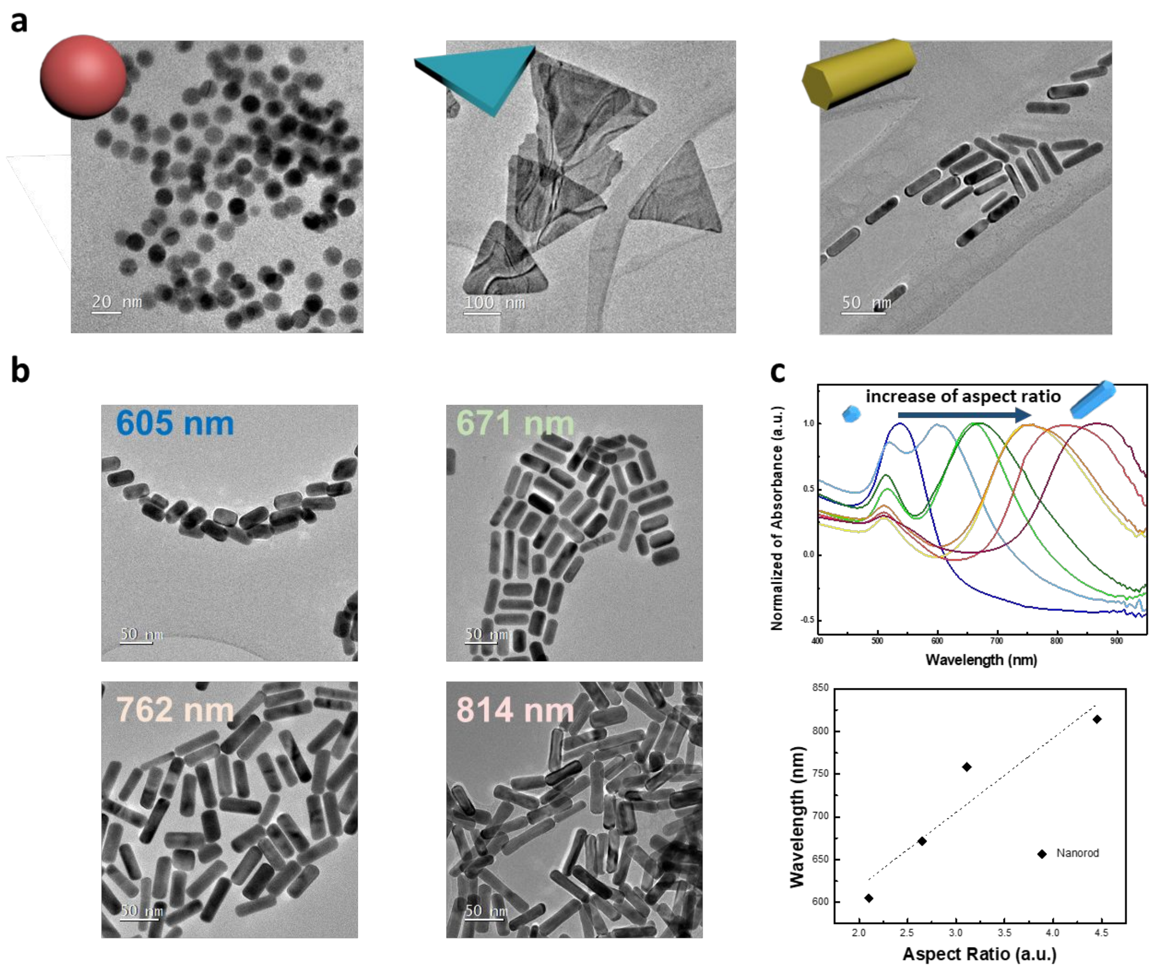

Supplementary Figure 13. Tunable size and shapes of the synthesized units for resonance control.

TEM image of the synthesized single-crystal Au nanostructures with a) tunable shapes b) tunable aspect ratio for controllable resonance. c) Absorption spectrum of the normalized absorption peaks of the synthesized gold nanorods with dependence on the aspect ratio.

S15. Setup and details of optical measurements

In this study, optical measurements were conducted using a home-built confocal microscope. The experimental setup and detailed information for single-point photoluminescence (PL) measurement are depicted in Supplementary Fig. 14. Optical emissions from the samples in large areas were verified by mapping point-by-point spectra with a step size of 150 nm. The excitation laser used was a continuous wave (CW) laser with a

wavelength of 532 nm. The laser beam was focused onto the sample surface with normal incidence. The PL signal was collected using a 100× objective lens with a numerical aperture (NA) of 0.9, a focal length of 1 mm, and a spatial resolution of approximately 500 nm. PL spectra were acquired using an Andor spectrometer with a measured spectral resolution of 0.3 nm and a cooled charge-coupled camera.

To study the second-order photon correlation function, a free-space Hanbury Brown and Twist interferometer was employed. Time-resolved photon counting was performed using two single-photon avalanche diodes (SPADs). No filters or background corrections were applied to spectrally select a single quantum emitter. Time-resolved PL measurements were carried out using a supercontinuum pulsed laser (NKT SuperK Extreme) with a pulse width of 5 ps, a repetition rate of 78 MHz, and a wavelength of 532 nm.

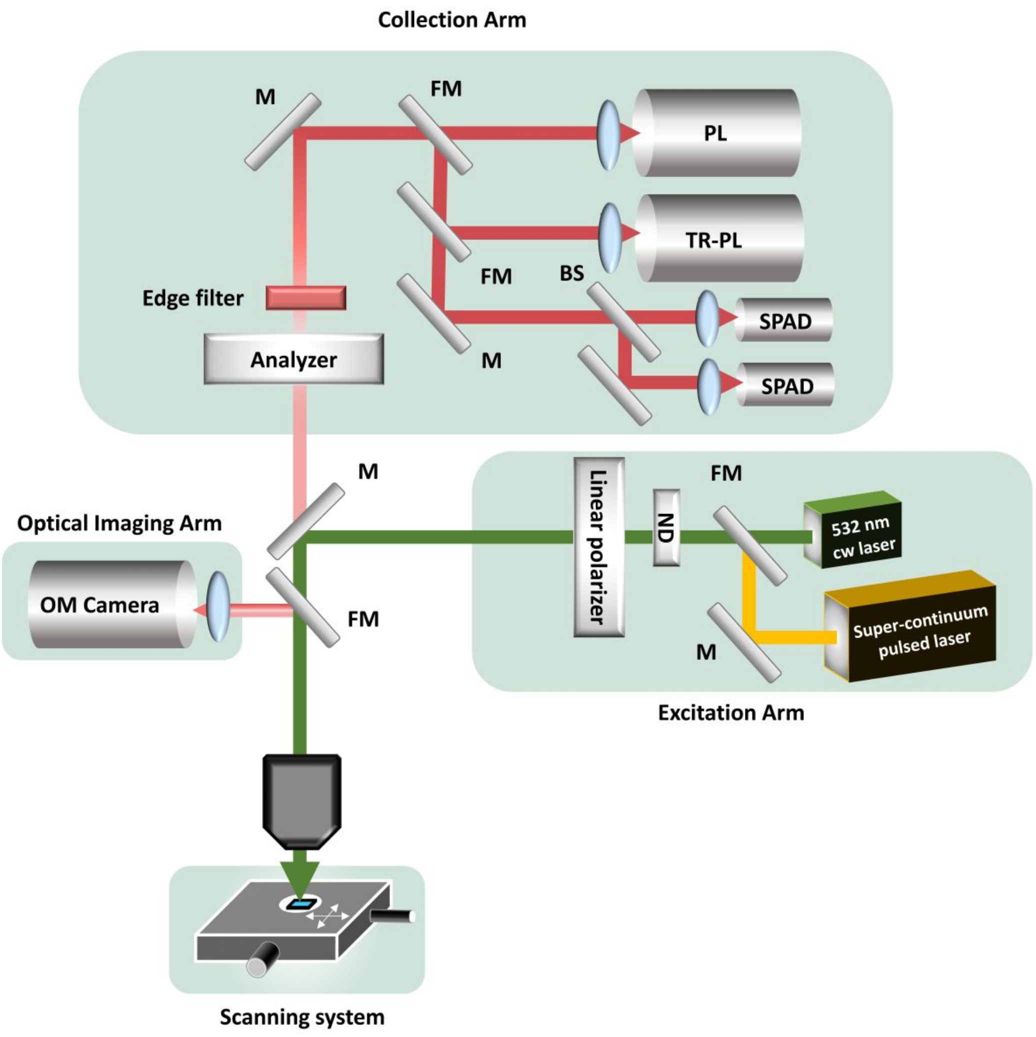

Supplementary Figure 14. Setup and details of optical measurements.

ND: Neutral-density filter. M: Mirror. FM: Flip Mirror. BS: Beam splitter. TR-PL: Time-resolved photoluminescence. SPAD: Single Photon Avalanche Diode.

S16. Reference

1. Zheng, Y.; Zhong, X.; Li, Z.; Xia, Y. Successive, Seed-Mediated Growth for the Synthesis of Single-Crystal Gold Nanospheres with Uniform Diameters Controlled in the Range of 5–150 nm. *Part. Part. Syst. Charact.* **2014**, *31* (2), 266-273.
2. Yang, T.-H.; Zhou, S.; Gilroy, K. D.; Figueroa-Cosme, L.; Lee, Y.-H.; Wu, J.-M.; Xia, Y. Autocatalytic surface reduction and its role in controlling seed-mediated growth of colloidal metal nanocrystals. *Proc. Natl. Acad. Sci. U.S.A.* **2017**, *114* (52), 13619-13624.
3. Yang, T.-H.; Peng, H.-C.; Zhou, S.; Lee, C.-T.; Bao, S.; Lee, Y.-H.; Wu, J.-M.; Xia, Y. Toward a Quantitative Understanding of the Reduction Pathways of a Salt Precursor in the Synthesis of Metal Nanocrystals. *Nano Lett.* **2017**, *17* (1), 334-340.
4. Derkachova, A., Kolwas, K. & Demchenko, I. Dielectric Function for Gold in Plasmonics Applications: Size Dependence of Plasmon Resonance Frequencies and Damping Rates for Nanospheres. *Plasmonics* **11***,* 941-951 (2016).
5. Liu, D.*, et al.* Rapid Synthesis of Monodisperse Au Nanospheres through a Laser Irradiation -Induced Shape Conversion, Self-Assembly and Their Electromagnetic Coupling SERS Enhancement. *Sci. Rep.* **5***,* 7686 (2015).
6. Mendelson, N.*, et al.* Engineering and Tuning of Quantum Emitters in Few-Layer Hexagonal Boron Nitride. *ACS Nano* **13***,* 3132-3140 (2019).
7. Tran, T.T., Bray, K., Ford, M.J., Toth, M. & Aharonovich, I. Quantum emission from hexagonal boron nitride monolayers. *Nat. Nanotechnol.* **11***,* 37-41 (2016).
8. Jeon, H.B., Tsalu, P.V. & Ha, J.W. Shape Effect on the Refractive Index Sensitivity at Localized Surface Plasmon Resonance Inflection Points of Single Gold Nanocubes with Vertices. *Sci. Rep.* **9***,* 13635 (2019).
